# 4-[2-(4-Chloro­phen­yl)hydrazinyl­idene]-3-methyl-5-oxo-4,5-dihydro-1*H*-pyrazole-1-carbothio­amide

**DOI:** 10.1107/S1600536811038463

**Published:** 2011-09-30

**Authors:** Hoong-Kun Fun, Suhana Arshad, Shobhitha Shetty, Balakrishna Kalluraya

**Affiliations:** a X-ray Crystallography Unit, School of Physics, Universiti Sains Malaysia, 11800 USM, Penang, Malaysia; b Department of Studies in Chemistry, Mangalore University, Mangalagangothri 574 199, Karnataka, India

## Abstract

In the title mol­ecule, C_11_H_10_ClN_5_OS, an intra­molecular N—H⋯O hydrogen forms an *S*(6) ring motif. The dihedral angle between the pyrazole ring and the benzene ring is 3.77 (8)°. In the crystal, mol­ecules are linked by N—H⋯S and N—H⋯O hydrogen bonds into layers parallel to the *bc* plane.

## Related literature

For the biological activity and pharmacological properties of pyrazole derivatives, see: Rai *et al.* (2008 ▶); Girisha *et al.* (2010 ▶); Isloor *et al.* (2009 ▶). For standard bond-length data, see: Allen *et al.* (1987 ▶). For hydrogen-bond motifs, see: Bernstein *et al.* (1995 ▶).
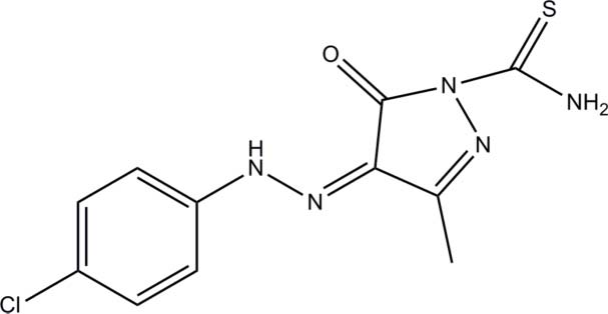


## Experimental

### 

#### Crystal data

C_11_H_10_ClN_5_OS
*M* *_r_* = 295.75
Monoclinic, 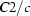

*a* = 25.0899 (17) Å
*b* = 11.6075 (9) Å
*c* = 9.0806 (6) Å
β = 99.516 (1)°
*V* = 2608.2 (3) Å^3^
*Z* = 8
Mo *K*α radiation
μ = 0.45 mm^−1^
*T* = 296 K
0.48 × 0.33 × 0.17 mm

#### Data collection

Bruker SMART APEXII DUO CCD area-detector diffractometer
Absorption correction: multi-scan (*SADABS*; Bruker, 2009 ▶) *T*_min_ = 0.812, *T*_max_ = 0.927
22139 measured reflections
3827 independent reflections
3125 reflections with *I* > 2σ(*I*)
*R*_int_ = 0.024

#### Refinement

*R*[*F* ^2^ > 2σ(*F* ^2^)] = 0.038
*wR*(*F* ^2^) = 0.120
*S* = 1.04
3827 reflections
185 parameters
H atoms treated by a mixture of independent and constrained refinement
Δρ_max_ = 0.36 e Å^−3^
Δρ_min_ = −0.49 e Å^−3^

### 

Data collection: *APEX2* (Bruker, 2009 ▶); cell refinement: *SAINT* (Bruker, 2009 ▶); data reduction: *SAINT*; program(s) used to solve structure: *SHELXTL* (Sheldrick, 2008 ▶); program(s) used to refine structure: *SHELXTL*; molecular graphics: *SHELXTL*; software used to prepare material for publication: *SHELXTL* and *PLATON* (Spek, 2009 ▶).

## Supplementary Material

Crystal structure: contains datablock(s) global, I. DOI: 10.1107/S1600536811038463/lh5337sup1.cif

Structure factors: contains datablock(s) I. DOI: 10.1107/S1600536811038463/lh5337Isup2.hkl

Supplementary material file. DOI: 10.1107/S1600536811038463/lh5337Isup3.cml

Additional supplementary materials:  crystallographic information; 3D view; checkCIF report

## Figures and Tables

**Table 1:** Hydrogen-bond geometry (Å, °)

*D*—H⋯*A*	*D*—H	H⋯*A*	*D*⋯*A*	*D*—H⋯*A*
N4—H1*N*4⋯O1	0.914 (18)	2.114 (19)	2.7903 (16)	129.9 (16)
N5—H1*N*5⋯S1^i^	0.89 (2)	2.76 (2)	3.5239 (13)	144.5 (16)
N5—H2*N*5⋯O1^ii^	0.91 (2)	2.00 (2)	2.9124 (15)	177.0 (19)

Symmetry codes: (i) 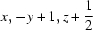; (ii) 
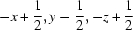
.

**Fig. 1.**
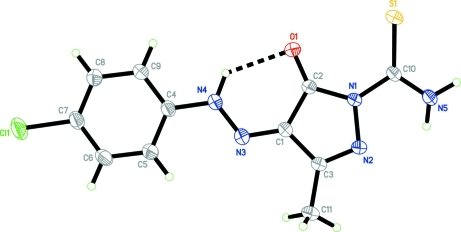
The molecular structure of the title compound, showing 30% probability displacement ellipsoids. The dashed line indicates an intramolecular bond.

**Fig. 2.**
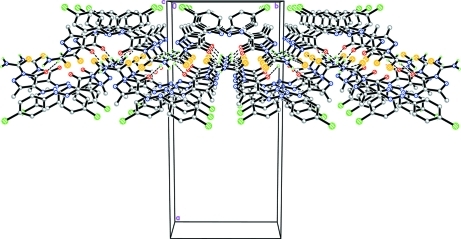
The crystal packing of the title compound. The dashed lines represent hydrogen bonds.

## supplementary crystallographic information

### Comment

Pyrazole derivatives are well established in the literatures as important biologically active heterocyclic compounds (Rai *et al.*, 2008). These derivatives are the subject of many research studies due to their widespread pharmacological properties such as anti-inflammatory (Girisha *et al.*, 2010), antipyretic, antimicrobial (Isloor *et al.*, 2009), and antiviral activities. The widely prescribed anti-inflammatory pyrazole derivatives, celecoxib and deracoxib, are selective COX-2 inhibitors with reduced ulcerogenic side effects. The synthetic route followed for obtaining the title compound involves the diazotization of substituted anilines to give the diazonium salts followed by coupling with ethyl acetoacetate in the presence of sodium acetate to give corresponding oxobutanoate which on further reaction with thiosemicarbazide in acetic acid gave the required thioamides.

The molecular structure is shown in Fig. 1. An intramolecular N4—H1N4···O1 hydrogen bond (Table 1) stabilizes the molecular structure and forms an *S*(6) ring motif (Bernstein *et al.*, 1995). The dihedral angle between the 4,5-dihydro-1*H*-pyrazole (N1/N2/C1–C3) ring and the phenyl (C4–C9) ring is 3.77 (8)°. Bond lengths (Allen *et al.*, 1987) and angles are within normal ranges.

The crystal packing is shown in Fig. 2. The molecules are linked by intermolecular N5—H1N5···S1^i^ and N5—H2N5···O1^ii^ hydrogen bonds (Table 1) into layers parallel to *bc* plane.

### Experimental

To a solution of ethyl-2-[(4-chlorophenyl)hydrazono]-3-oxobutanoate (0.01 mol) dissolved in glacial acetic acid (20 ml), a solution of thiosemicarbazide (0.02 mol) in glacial acetic acid (25 ml) was added and the mixture was refluxed for 4 h. It was cooled and allowed to stand overnight. The solid product that separated out was filtered and dried. It was then recrystallized from ethanol. Crystals suitable for X-ray analysis were obtained from 1:2 mixtures of DMF and ethanol by slow evaporation.

### Refinement

N-bound H atoms was located from the difference map and refined freely, [N–H = 0.89 (2)–0.912 (18) Å]. The remaining H atoms were positioned geometrically [C–H = 0.93 or 0.96 Å] and refined using a riding model with *U*_iso_(H) = 1.2 or 1.5 *U*_eq_(C). A rotating group model was applied to the methyl group.

### Crystal data

**Table:**

C_11_H_10_ClN_5_OS	*F*(000) = 1216
*M**_r_* = 295.75	*D*_x_ = 1.506 Mg m^−^^3^
Monoclinic, *C*2/*c*	Mo *K*α radiation, λ = 0.71073 Å
Hall symbol: -C 2yc	Cell parameters from 9651 reflections
*a* = 25.0899 (17) Å	θ = 2.9–29.9°
*b* = 11.6075 (9) Å	µ = 0.45 mm^−^^1^
*c* = 9.0806 (6) Å	*T* = 296 K
β = 99.516 (1)°	Block, orange
*V* = 2608.2 (3) Å^3^	0.48 × 0.33 × 0.17 mm
*Z* = 8	

### Data collection

**Table:**

Bruker SMART APEXII DUO CCD area-detector diffractometer	3827 independent reflections
Radiation source: fine-focus sealed tube	3125 reflections with *I* > 2σ(*I*)
graphite	*R*_int_ = 0.024
φ and ω scans	θ_max_ = 30.1°, θ_min_ = 1.7°
Absorption correction: multi-scan (*SADABS*; Bruker, 2009)	*h* = −35→35
*T*_min_ = 0.812, *T*_max_ = 0.927	*k* = −16→16
22139 measured reflections	*l* = −12→12

### Refinement

**Table:**

Refinement on *F*^2^	Primary atom site location: structure-invariant direct methods
Least-squares matrix: full	Secondary atom site location: difference Fourier map
*R*[*F*^2^ > 2σ(*F*^2^)] = 0.038	Hydrogen site location: inferred from neighbouring sites
*wR*(*F*^2^) = 0.120	H atoms treated by a mixture of independent and constrained refinement
*S* = 1.04	*w* = 1/[σ^2^(*F*_o_^2^) + (0.0678*P*)^2^ + 1.0988*P*] where *P* = (*F*_o_^2^ + 2*F*_c_^2^)/3
3827 reflections	(Δ/σ)_max_ = 0.003
185 parameters	Δρ_max_ = 0.36 e Å^−^^3^
0 restraints	Δρ_min_ = −0.49 e Å^−^^3^

### Special details

**Table:**

Geometry. All e.s.d.'s (except the e.s.d. in the dihedral angle between two l.s. planes) are estimated using the full covariance matrix. The cell e.s.d.'s are taken into account individually in the estimation of e.s.d.'s in distances, angles and torsion angles; correlations between e.s.d.'s in cell parameters are only used when they are defined by crystal symmetry. An approximate (isotropic) treatment of cell e.s.d.'s is used for estimating e.s.d.'s involving l.s. planes.
Refinement. Refinement of *F*^2^ against ALL reflections. The weighted *R*-factor *wR* and goodness of fit *S* are based on *F*^2^, conventional *R*-factors *R* are based on *F*, with *F* set to zero for negative *F*^2^. The threshold expression of *F*^2^ > σ(*F*^2^) is used only for calculating *R*-factors(gt) *etc*. and is not relevant to the choice of reflections for refinement. *R*-factors based on *F*^2^ are statistically about twice as large as those based on *F*, and *R*- factors based on ALL data will be even larger.

### Fractional atomic coordinates and isotropic or equivalent isotropic displacement parameters (Å^2^)

**Table:**

	*x*	*y*	*z*	*U*_iso_*/*U*_eq_	
S1	0.247690 (16)	0.67094 (3)	0.16327 (4)	0.04930 (13)	
Cl1	0.47498 (2)	1.36700 (4)	0.93948 (6)	0.07500 (18)	
O1	0.29887 (5)	0.85770 (9)	0.39231 (11)	0.0481 (3)	
N1	0.30587 (5)	0.65709 (9)	0.43842 (11)	0.0376 (2)	
N2	0.33273 (5)	0.58737 (10)	0.55643 (12)	0.0456 (3)	
N3	0.37848 (5)	0.86126 (10)	0.67837 (13)	0.0431 (3)	
N4	0.36803 (5)	0.96471 (10)	0.62440 (13)	0.0424 (3)	
N5	0.26388 (6)	0.49214 (11)	0.34633 (13)	0.0489 (3)	
C1	0.35437 (5)	0.77370 (11)	0.60545 (14)	0.0397 (3)	
C2	0.31655 (5)	0.77385 (11)	0.46481 (13)	0.0358 (2)	
C3	0.36083 (7)	0.65570 (12)	0.65135 (16)	0.0471 (3)	
C4	0.39475 (5)	1.05982 (11)	0.69841 (14)	0.0389 (3)	
C5	0.43223 (6)	1.04497 (13)	0.82750 (17)	0.0504 (3)	
H5A	0.4408	0.9714	0.8647	0.060*	
C6	0.45677 (7)	1.14060 (14)	0.90042 (19)	0.0557 (4)	
H6A	0.4818	1.1317	0.9875	0.067*	
C7	0.44409 (6)	1.24900 (13)	0.84375 (17)	0.0479 (3)	
C8	0.40700 (6)	1.26442 (13)	0.71486 (17)	0.0510 (3)	
H8A	0.3988	1.3380	0.6774	0.061*	
C9	0.38221 (6)	1.16883 (12)	0.64217 (17)	0.0473 (3)	
H9A	0.3571	1.1780	0.5554	0.057*	
C10	0.27250 (5)	0.60195 (11)	0.32065 (13)	0.0374 (3)	
C11	0.39486 (10)	0.61297 (16)	0.7902 (2)	0.0773 (6)	
H11A	0.3845	0.5356	0.8097	0.116*	
H11B	0.3899	0.6616	0.8724	0.116*	
H11C	0.4322	0.6141	0.7782	0.116*	
H1N4	0.3454 (7)	0.9766 (17)	0.536 (2)	0.052 (5)*	
H1N5	0.2758 (8)	0.4615 (18)	0.435 (2)	0.060 (5)*	
H2N5	0.2431 (8)	0.4515 (19)	0.272 (2)	0.064 (6)*	

### Atomic displacement parameters (Å^2^)

**Table:**

	*U*^11^	*U*^22^	*U*^33^	*U*^12^	*U*^13^	*U*^23^
S1	0.0633 (2)	0.0481 (2)	0.03108 (17)	0.00100 (15)	−0.00811 (14)	0.00135 (12)
Cl1	0.0730 (3)	0.0509 (2)	0.0885 (4)	−0.00312 (19)	−0.0239 (2)	−0.0250 (2)
O1	0.0625 (6)	0.0356 (5)	0.0409 (5)	0.0050 (4)	−0.0071 (4)	0.0029 (4)
N1	0.0460 (6)	0.0343 (5)	0.0287 (5)	−0.0019 (4)	−0.0046 (4)	0.0015 (4)
N2	0.0584 (7)	0.0352 (5)	0.0371 (5)	−0.0016 (5)	−0.0104 (5)	0.0047 (4)
N3	0.0486 (6)	0.0387 (5)	0.0392 (5)	−0.0032 (5)	−0.0011 (5)	−0.0034 (4)
N4	0.0485 (6)	0.0366 (5)	0.0384 (5)	−0.0032 (4)	−0.0034 (5)	−0.0035 (4)
N5	0.0674 (8)	0.0416 (6)	0.0326 (5)	−0.0111 (6)	−0.0067 (5)	−0.0016 (5)
C1	0.0455 (7)	0.0359 (6)	0.0343 (5)	−0.0020 (5)	−0.0035 (5)	−0.0011 (5)
C2	0.0411 (6)	0.0342 (6)	0.0306 (5)	0.0010 (5)	0.0019 (4)	−0.0003 (4)
C3	0.0566 (8)	0.0391 (6)	0.0389 (6)	−0.0021 (6)	−0.0118 (6)	0.0031 (5)
C4	0.0398 (6)	0.0382 (6)	0.0374 (6)	−0.0020 (5)	0.0020 (5)	−0.0059 (5)
C5	0.0521 (8)	0.0413 (7)	0.0515 (8)	0.0038 (6)	−0.0099 (6)	−0.0034 (6)
C6	0.0529 (8)	0.0523 (8)	0.0531 (8)	0.0040 (7)	−0.0175 (7)	−0.0083 (7)
C7	0.0438 (7)	0.0429 (7)	0.0529 (7)	−0.0011 (5)	−0.0037 (6)	−0.0127 (6)
C8	0.0552 (8)	0.0379 (7)	0.0547 (8)	−0.0012 (6)	−0.0060 (6)	−0.0025 (6)
C9	0.0518 (8)	0.0412 (7)	0.0431 (7)	−0.0022 (6)	−0.0087 (6)	−0.0011 (5)
C10	0.0414 (6)	0.0404 (6)	0.0286 (5)	−0.0022 (5)	0.0008 (4)	−0.0036 (4)
C11	0.1033 (15)	0.0516 (9)	0.0583 (10)	−0.0039 (9)	−0.0415 (10)	0.0100 (8)

### Geometric parameters (Å, °)

**Table:**

S1—C10	1.6664 (13)	C1—C2	1.4597 (17)
Cl1—C7	1.7347 (14)	C3—C11	1.486 (2)
O1—C2	1.2169 (16)	C4—C9	1.3813 (19)
N1—C2	1.3945 (16)	C4—C5	1.3870 (19)
N1—C10	1.3995 (15)	C5—C6	1.384 (2)
N1—N2	1.4207 (15)	C5—H5A	0.9300
N2—C3	1.2916 (18)	C6—C7	1.377 (2)
N3—C1	1.3057 (17)	C6—H6A	0.9300
N3—N4	1.3070 (16)	C7—C8	1.381 (2)
N4—C4	1.4039 (16)	C8—C9	1.385 (2)
N4—H1N4	0.912 (18)	C8—H8A	0.9300
N5—C10	1.3200 (18)	C9—H9A	0.9300
N5—H1N5	0.89 (2)	C11—H11A	0.9600
N5—H2N5	0.91 (2)	C11—H11B	0.9600
C1—C3	1.4331 (19)	C11—H11C	0.9600
			
C2—N1—C10	130.50 (11)	C6—C5—H5A	120.3
C2—N1—N2	111.74 (10)	C4—C5—H5A	120.3
C10—N1—N2	117.72 (10)	C7—C6—C5	119.82 (14)
C3—N2—N1	106.99 (11)	C7—C6—H6A	120.1
C1—N3—N4	118.53 (12)	C5—C6—H6A	120.1
N3—N4—C4	119.51 (11)	C6—C7—C8	121.12 (13)
N3—N4—H1N4	121.7 (12)	C6—C7—Cl1	118.55 (11)
C4—N4—H1N4	118.7 (12)	C8—C7—Cl1	120.33 (12)
C10—N5—H1N5	120.4 (13)	C7—C8—C9	119.12 (14)
C10—N5—H2N5	117.2 (13)	C7—C8—H8A	120.4
H1N5—N5—H2N5	122.2 (19)	C9—C8—H8A	120.4
N3—C1—C3	125.15 (12)	C4—C9—C8	120.05 (13)
N3—C1—C2	128.49 (12)	C4—C9—H9A	120.0
C3—C1—C2	106.35 (11)	C8—C9—H9A	120.0
O1—C2—N1	129.93 (12)	N5—C10—N1	113.67 (11)
O1—C2—C1	126.90 (12)	N5—C10—S1	124.52 (10)
N1—C2—C1	103.17 (10)	N1—C10—S1	121.81 (10)
N2—C3—C1	111.70 (12)	C3—C11—H11A	109.5
N2—C3—C11	122.34 (14)	C3—C11—H11B	109.5
C1—C3—C11	125.96 (13)	H11A—C11—H11B	109.5
C9—C4—C5	120.50 (12)	C3—C11—H11C	109.5
C9—C4—N4	118.80 (12)	H11A—C11—H11C	109.5
C5—C4—N4	120.68 (12)	H11B—C11—H11C	109.5
C6—C5—C4	119.38 (14)		
			
C2—N1—N2—C3	−2.16 (17)	C2—C1—C3—C11	−178.78 (18)
C10—N1—N2—C3	−179.95 (13)	N3—N4—C4—C9	−178.31 (14)
C1—N3—N4—C4	−178.03 (13)	N3—N4—C4—C5	0.3 (2)
N4—N3—C1—C3	−178.84 (15)	C9—C4—C5—C6	0.5 (2)
N4—N3—C1—C2	1.4 (2)	N4—C4—C5—C6	−178.13 (14)
C10—N1—C2—O1	1.0 (2)	C4—C5—C6—C7	−0.5 (3)
N2—N1—C2—O1	−176.43 (14)	C5—C6—C7—C8	0.2 (3)
C10—N1—C2—C1	179.99 (13)	C5—C6—C7—Cl1	179.16 (14)
N2—N1—C2—C1	2.57 (15)	C6—C7—C8—C9	0.1 (3)
N3—C1—C2—O1	−3.2 (2)	Cl1—C7—C8—C9	−178.82 (13)
C3—C1—C2—O1	177.00 (14)	C5—C4—C9—C8	−0.2 (2)
N3—C1—C2—N1	177.80 (14)	N4—C4—C9—C8	178.48 (14)
C3—C1—C2—N1	−2.04 (15)	C7—C8—C9—C4	−0.2 (3)
N1—N2—C3—C1	0.72 (19)	C2—N1—C10—N5	−167.44 (14)
N1—N2—C3—C11	−179.63 (18)	N2—N1—C10—N5	9.85 (18)
N3—C1—C3—N2	−178.98 (14)	C2—N1—C10—S1	13.6 (2)
C2—C1—C3—N2	0.86 (19)	N2—N1—C10—S1	−169.10 (10)
N3—C1—C3—C11	1.4 (3)		

### Hydrogen-bond geometry (Å, °)

**Table:**

*D*—H···*A*	*D*—H	H···*A*	*D*···*A*	*D*—H···*A*
N4—H1N4···O1	0.914 (18)	2.114 (19)	2.7903 (16)	129.9 (16)
N5—H1N5···S1^i^	0.89 (2)	2.76 (2)	3.5239 (13)	144.5 (16)
N5—H2N5···O1^ii^	0.91 (2)	2.00 (2)	2.9124 (15)	177.0 (19)

Symmetry codes: (i) *x*, −*y*+1, *z*+1/2; (ii) −*x*+1/2, *y*−1/2, −*z*+1/2.
